# Supplementary figures and images for: Scalable multi-sample single-cell data analysis by Partition-Assisted Clustering and Multiple Alignments of Networks
Source: PLoS Comput Biol. 2017 Dec 27;13(12):e1005875. doi: 10.1371/journal.pcbi.1005875 (PMC5760091; doi:10.1371/journal.pcbi.1005875)

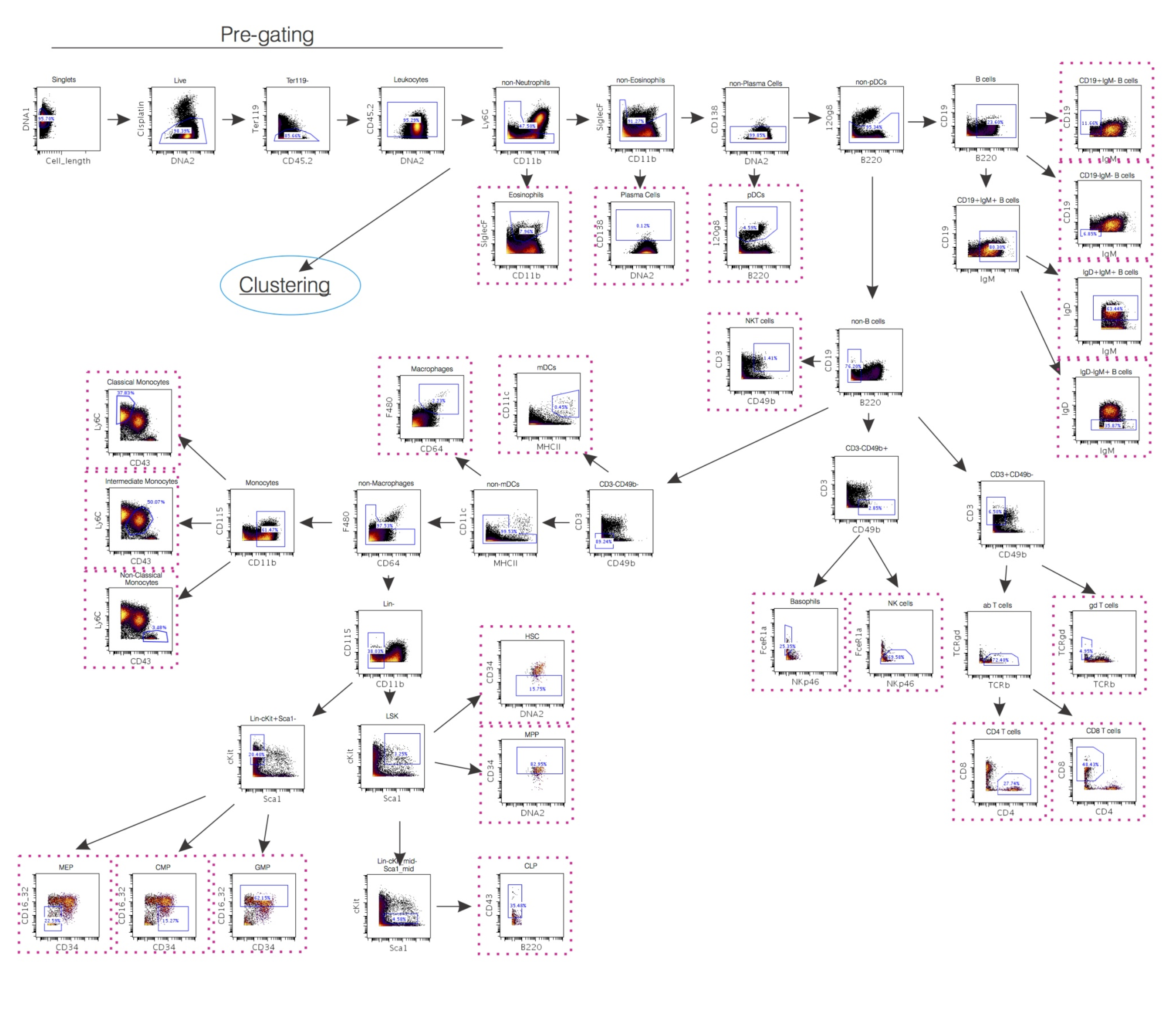

Supplement: S1 Fig — Biaxial gating hierarchy for the mouse bone marrow CyTOF dataset. Gating strategy that was used to find 24 reference populations in the mouse bone marrow CyTOF data. Pre-gating step involved removal of doublets, dead cells, erythrocytes and neutrophils. Non-neutrophils population was either subject to cluster analysis by computational tools or subsequent gating. Dotted boxes represent 24 terminal gates that were selected as reference populations for the comparison analysis. (TIF) [file pcbi.1005875.s001.tif]

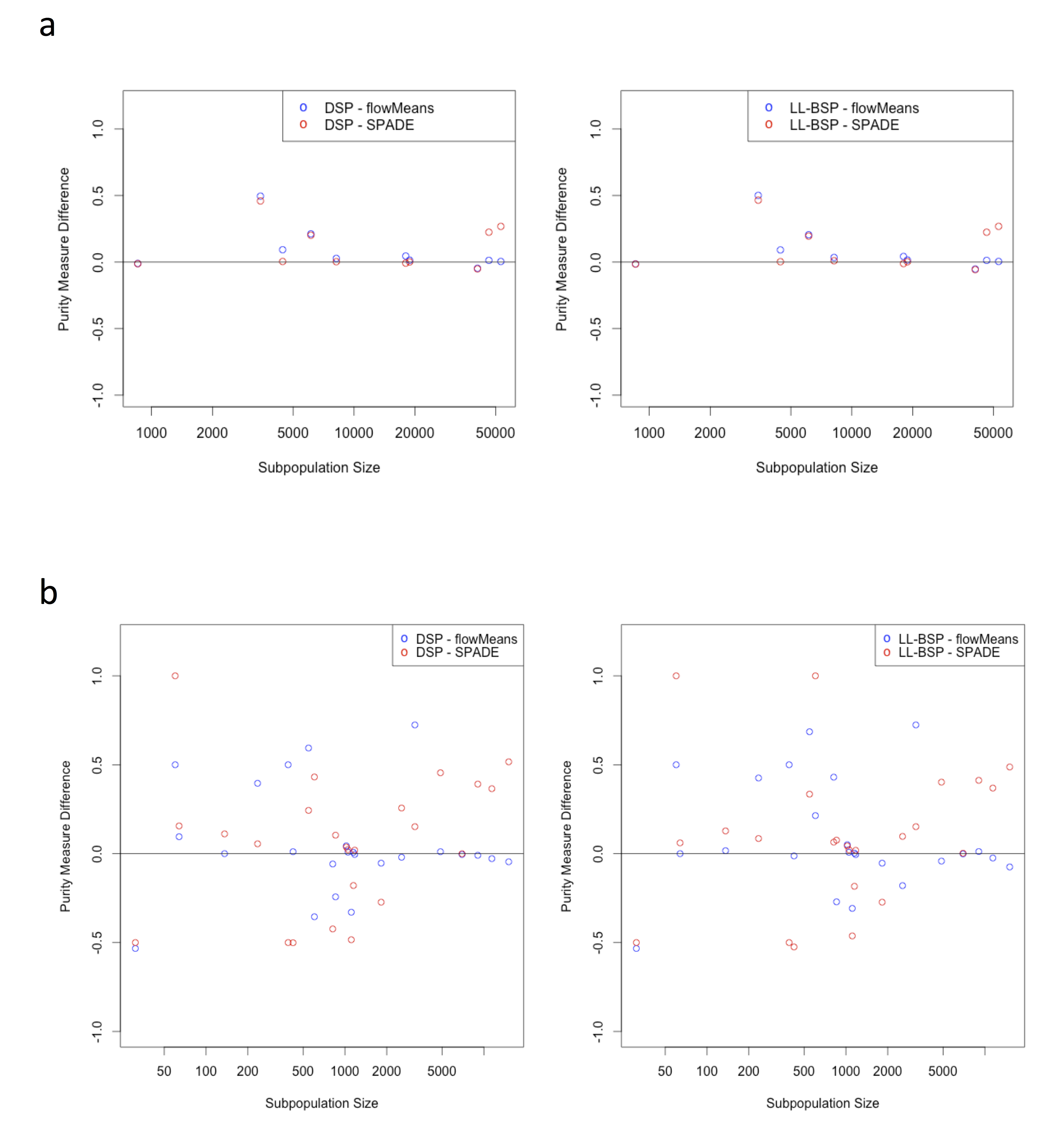

Supplement: S2 Fig — (a) Subpopulation-specific purity plot of 35-dimensional simulated data with 10 subpopulations. The blue points denote the differences between the p-measures of the partition-based method (either d-PAC or b-PAC) and flowMeans, while the red points denote the p-measure differences between the partition methods and SPADE. The horizontal line at 0 means no difference between the methods. Most of the blue and red points are above 0, indicating that the PAC generates purer subpopulations compared to the ground truth. The two subplots are very similar, which means that d-PAC and b-PAC give very similar p-measures. More precisely, the sum of differences between d-PAC and flowMeans and d-PAC and SPADE are 0.85 and 1.09, respectively; and the overall difference between b-PAC and flowMeans and b-PAC and SPADE are 0.84 and 1.08, respectively. (b) Subpopulation-specific purity plot of the hand-gated CyTOF data. The same convention is used as in (S2A Fig). Again, more blue and red points are above 0, indicating that the partition-based methods generate purer subpopulations compared to the ground truth. There is a cluster of points below 0 occurring in the middle of the plot, suggesting that flowMeans and SPADE capture the mid-size subpopulations more similar to hand-gating than the partition-based methods. More specifically, flowMeans does better (p-measure difference of 0.1 or better; difference of less 0.1 is considered practically no difference) with finding subpopulations of GMP, CD8 T cells, MEP, CD4 T cells (compared to d-PAC), and Plasma cells, while SPADE does better with CD19+IgM- B cells, NK cells (compared to d-PAC), CD8 T cells, NKT cells, Basophils, Short-Term HSC, and Plasma cells. However, overall, PAC has a much better performance, as the absolute sum of points above 0 is higher than that of points below 0. More precisely, the sum of differences between d-PAC and flowMeans and d-PAC and SPADE are 1.21 and 1.45, respectively; and the overall difference between b-P [file pcbi.1005875.s002.tif]

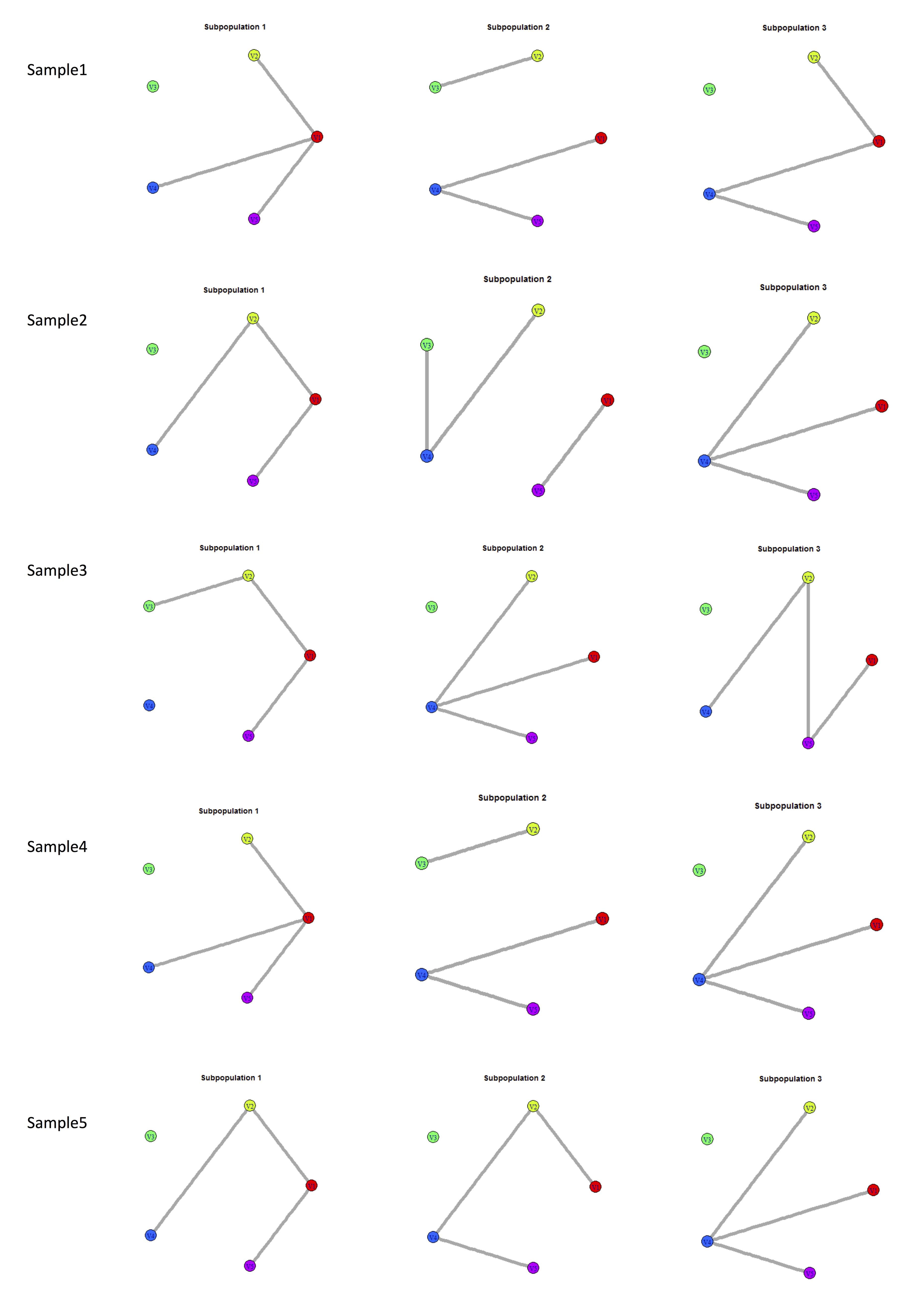

Supplement: S3 Fig — Fig 9 introduced the dynamic example in which five samples each having 2 true subpopulations captures the almost-convergence of means. Here the underlying network structures for the PAC discovered subpopulations (three per sample) in Fig 10 are presented. (TIF) [file pcbi.1005875.s003.tif]

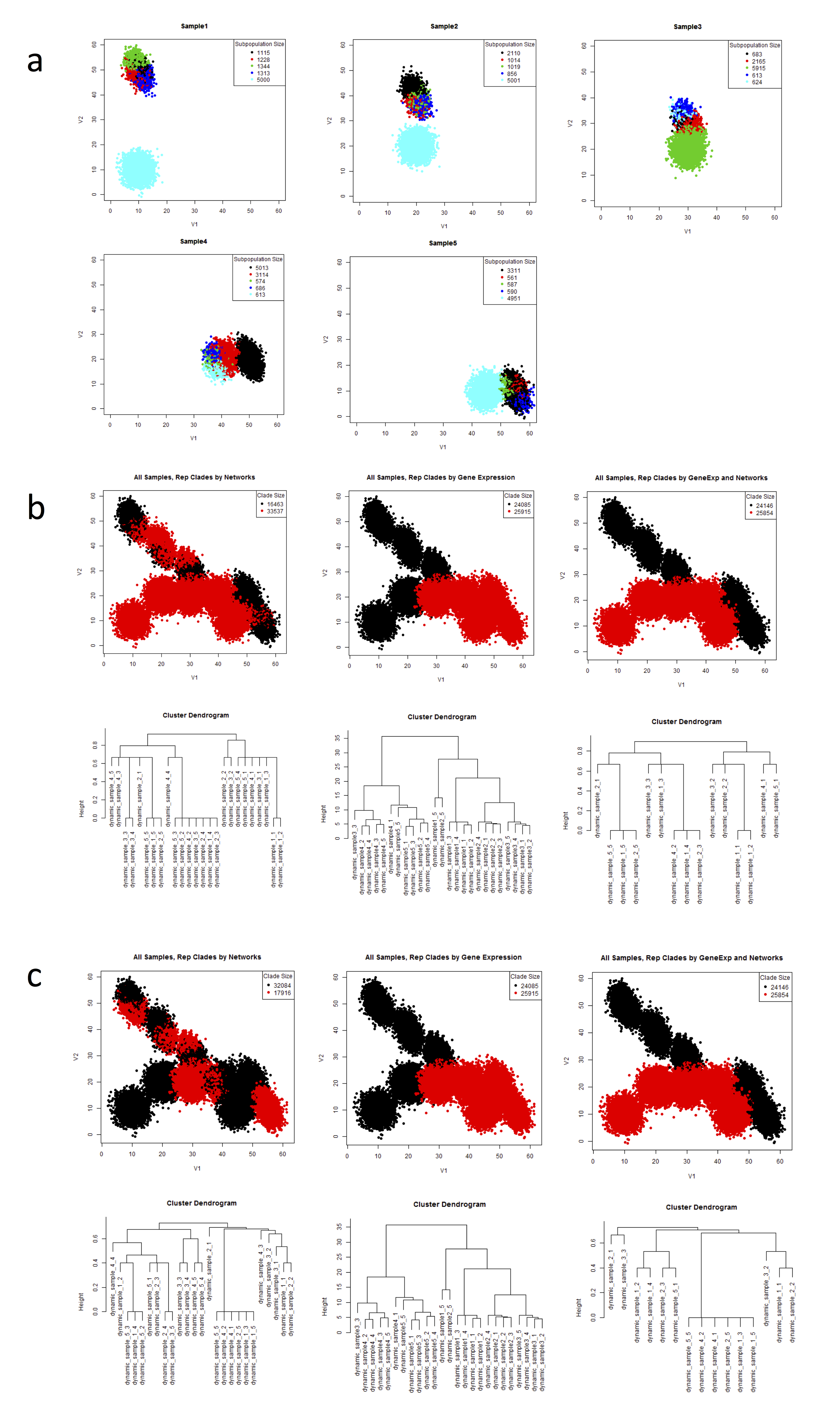

Supplement: S4 Fig — (a) PAC can be used to discover more subpopulations, with the effect of more partitions from the true clusters. (b) When over-partitioning is present, network or expression profile alone cannot resolve the dynamic (or batch) effects due to noisy covariance for small fragments of distributions. However, first aligning the larger subpopulations with more stable covariance, and thus network structures, and then merge in the smaller subpopulations by expression profile resolves the effects. (c) If more irrelevant edges were introduced, network alignment would fail due to the negative impact of the miscellaneous edges; however, eliminating small subpopulations from the alignment step alleviates the increased edge count problem. (TIF) [file pcbi.1005875.s004.tif]

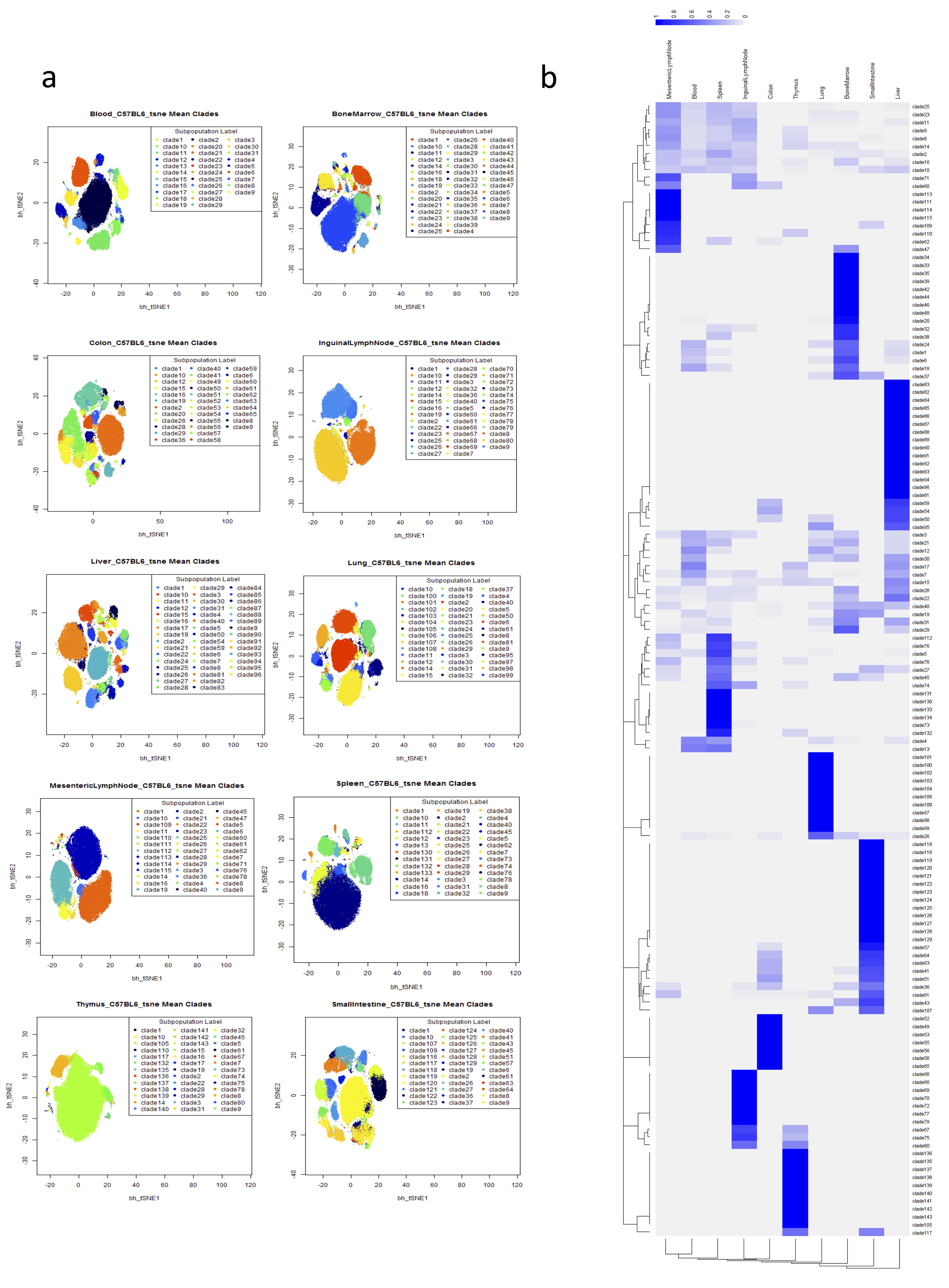

Supplement: S5 Fig — (a) t-SNE plots of mouse tissue samples colored by representative subpopulations labels from linkage by means. (b) Subpopulation proportion heatmap of clades of samples from linkage by means. (TIF) [file pcbi.1005875.s005.tif]

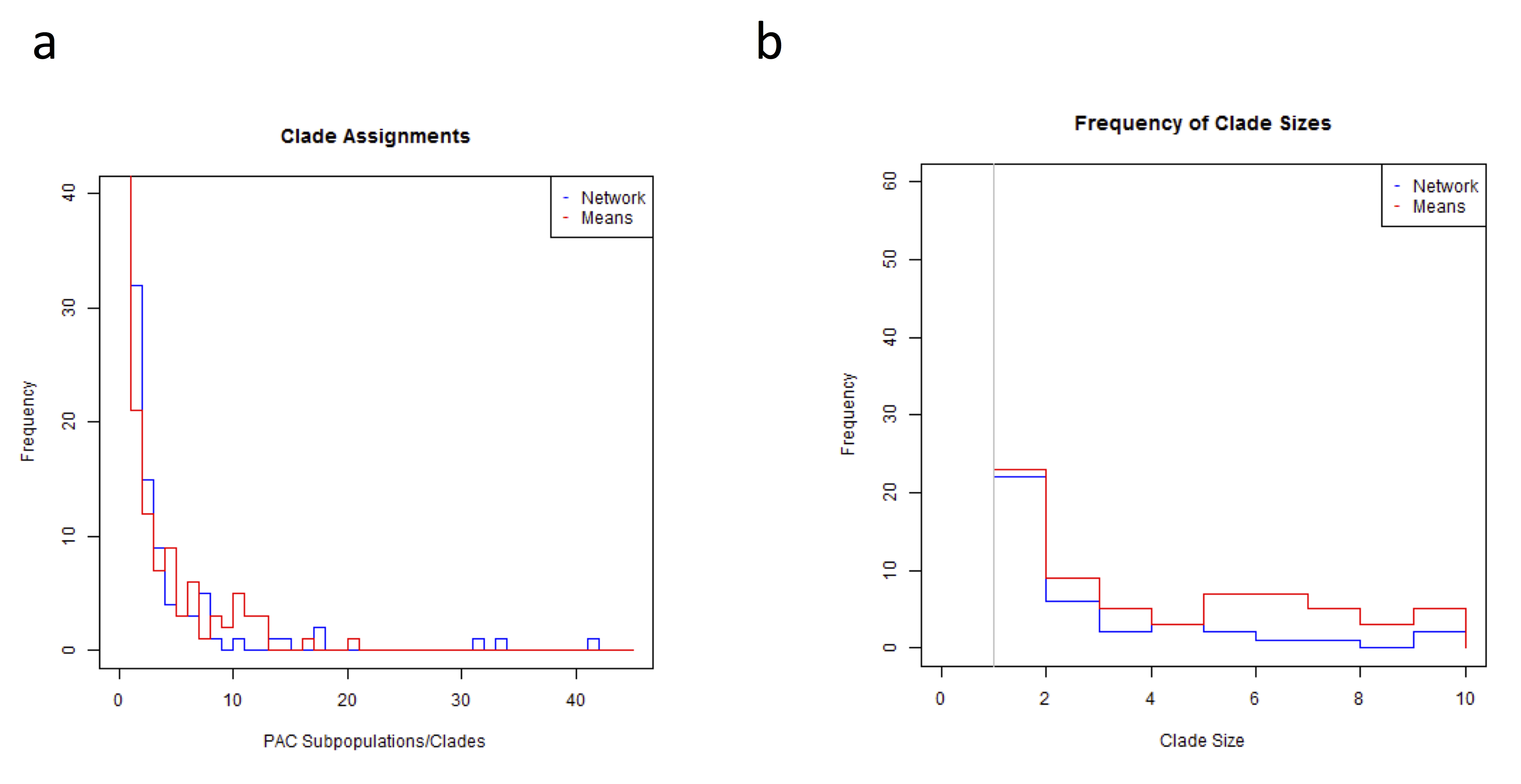

Supplement: S6 Fig — (a) PAC-discovered subpopulations are aggregated by MAN into clades; the number of PAC subpopulations/clades for the network and means PAC-MAN approaches are plotted. (b) After aggregating shared clades within samples, the number of shared clades for the entire dataset is plotted for the two PAC-MAN approaches. (TIF) [file pcbi.1005875.s006.tif]

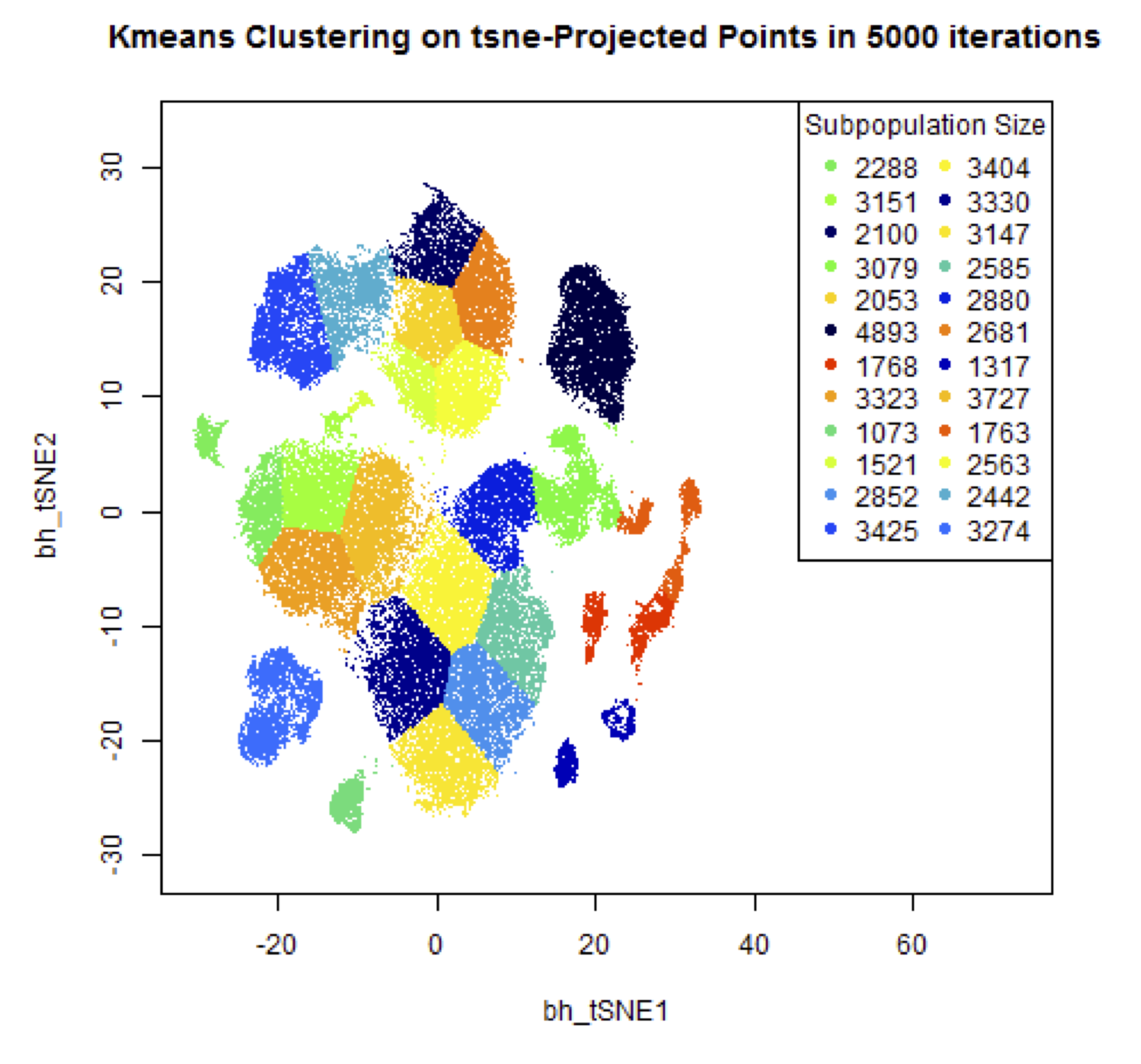

Supplement: S7 Fig — We use t-SNE plots heavily for visualization in our study. We tested the approach of clustering on t-SNE projected points using kmeans. We observe that, despite being a very valuable visualization tool, t-SNE points do not contain much information for defining well-separated clusters for the usual clustering algorithms that depend on Gaussian geometry. It is best to perform the clustering using all data points in the original high-dimensional space, and then use t-SNE to visualize a subset of the points (amount chosen with computational capacity to run t-SNE). (TIF) [file pcbi.1005875.s007.tif]

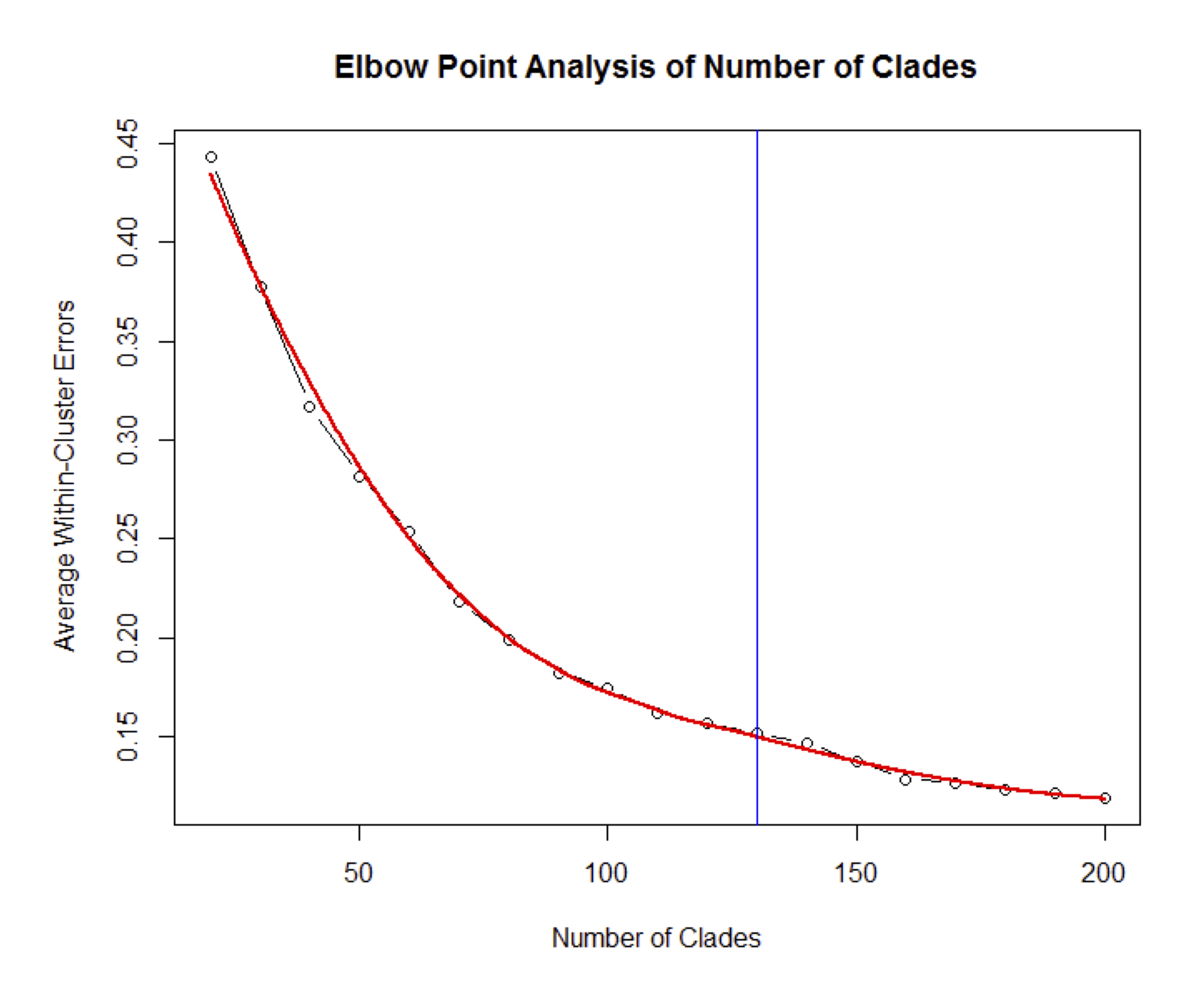

Supplement: S8 Fig — Elbow point analysis is the most computationally feasible approach to find the optimal number of clades to output. We calculated the within-cluster errors (from the centroid) for each of the example tissue sample. Next, we averaged the within-cluster errors for all 10 tissue samples. This calculation was performed for a range of numbers of clades. Next, loess smoothing was applied to the average within-cluster errors over the numbers of clades. The elbow point occurs at 130 clades, highlighted by the vertical blue line. (TIF) [file pcbi.1005875.s008.tif]

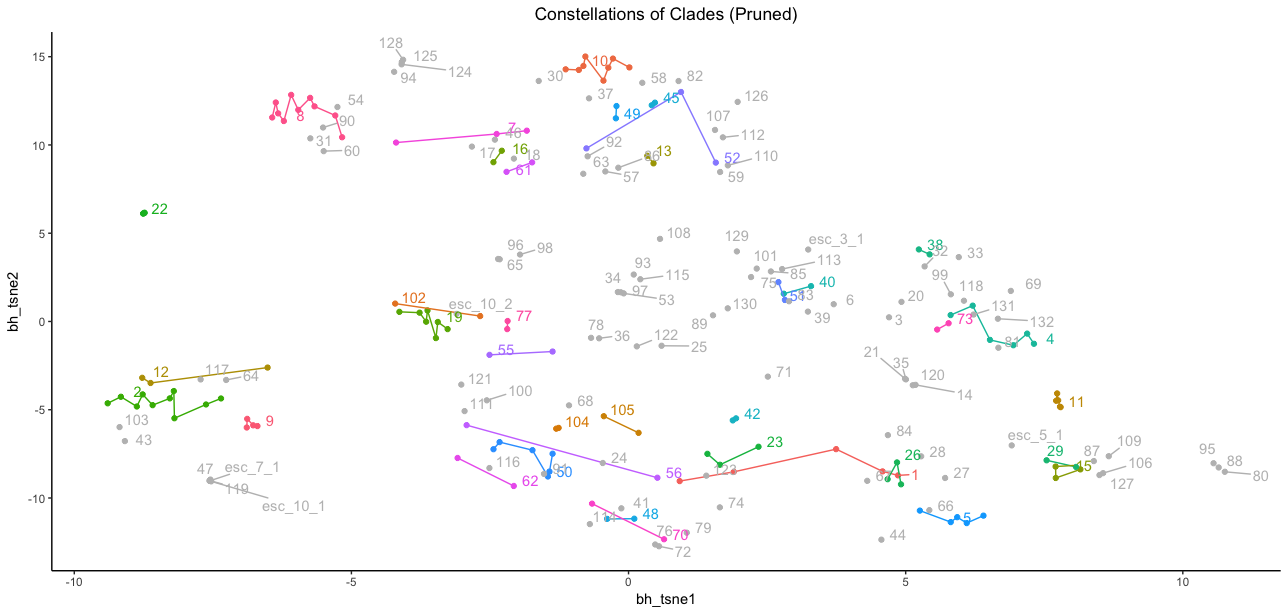

Supplement: S9 Fig — As descripted in Materials and Methods, relative distances between subpopulations and clade centers are utilized to prune away subpopulations that are far away within clades. Clades 6 and 39 were pruned by setting threshold constant multiplier at 2. (TIF) [file pcbi.1005875.s009.tif]

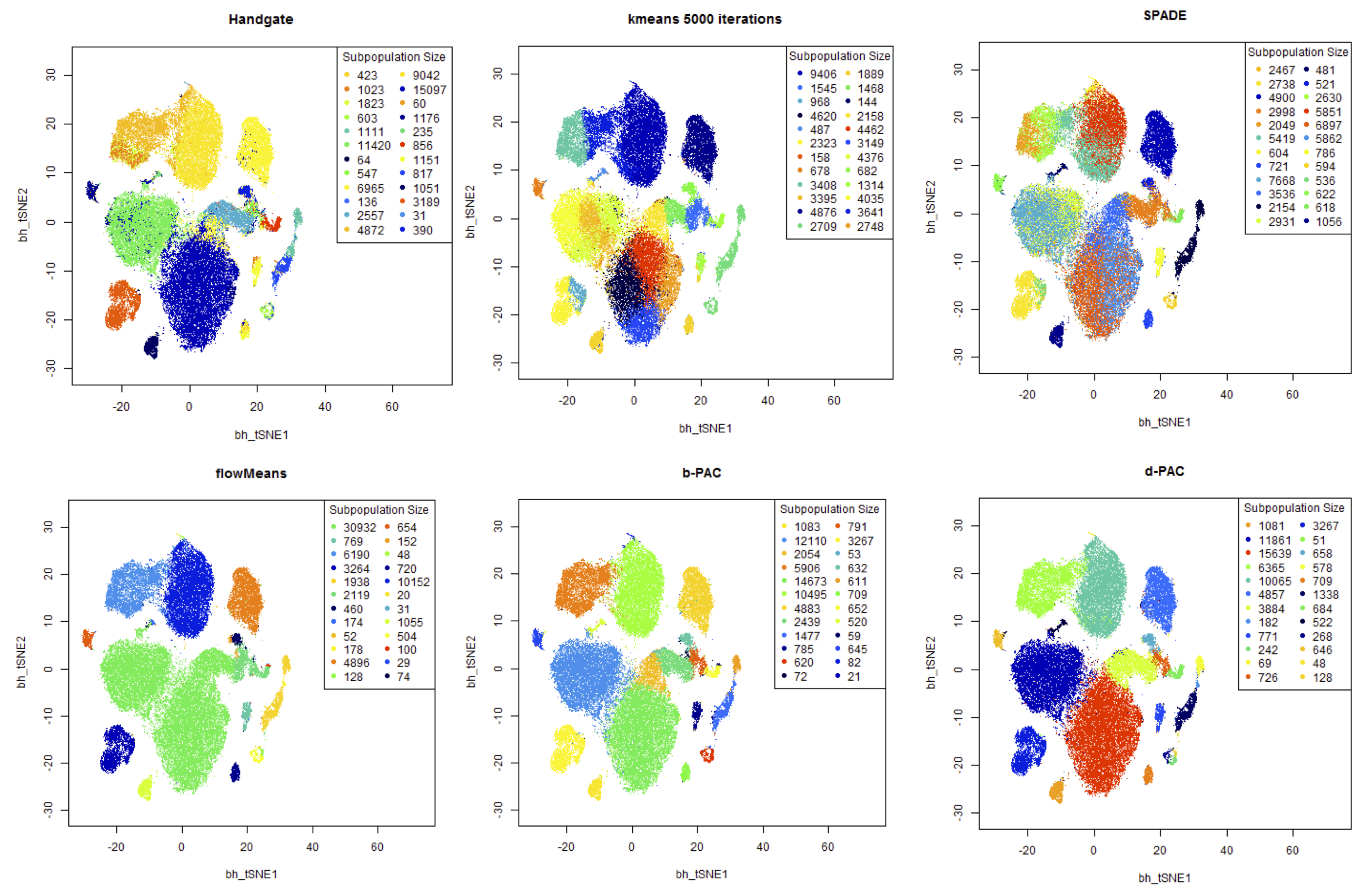

Supplement: S10 Fig — Higher resolution version of Fig 4. (TIFF) [file pcbi.1005875.s010.tiff]

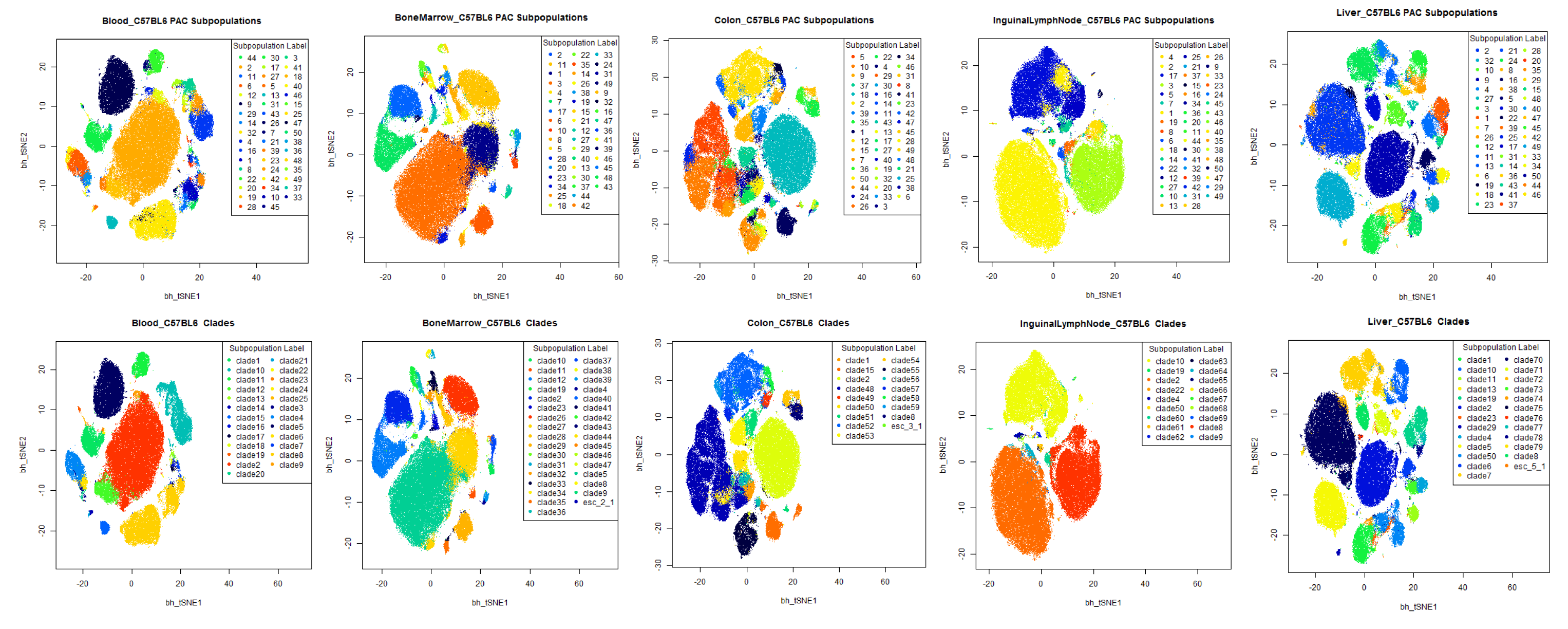

Supplement: S11 Fig — Higher resolution version of Fig 12 (in another color scheme) with subpopulation and clade labels. (TIF) [file pcbi.1005875.s011.tif]

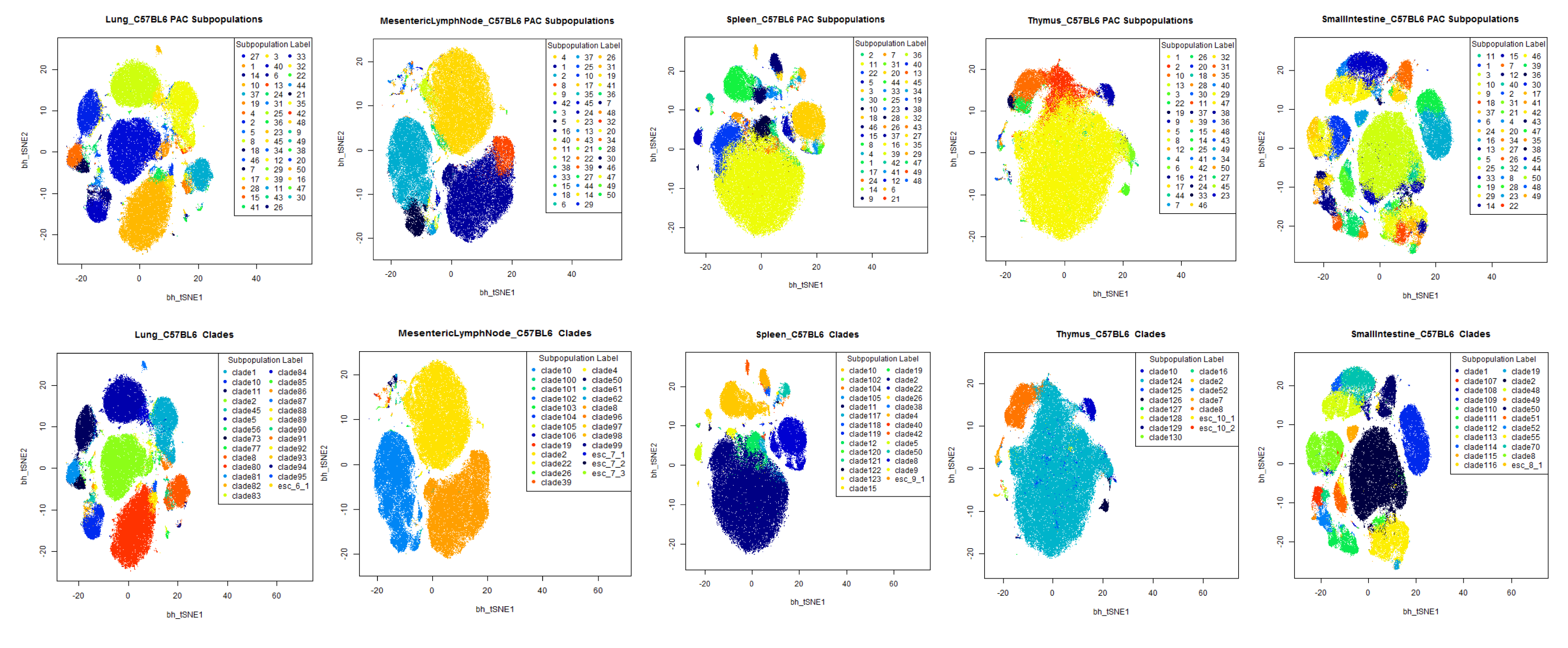

Supplement: S12 Fig — Higher resolution version of Fig 13 (in another color scheme) with subpopulation and clade labels. (TIF) [file pcbi.1005875.s012.tif]

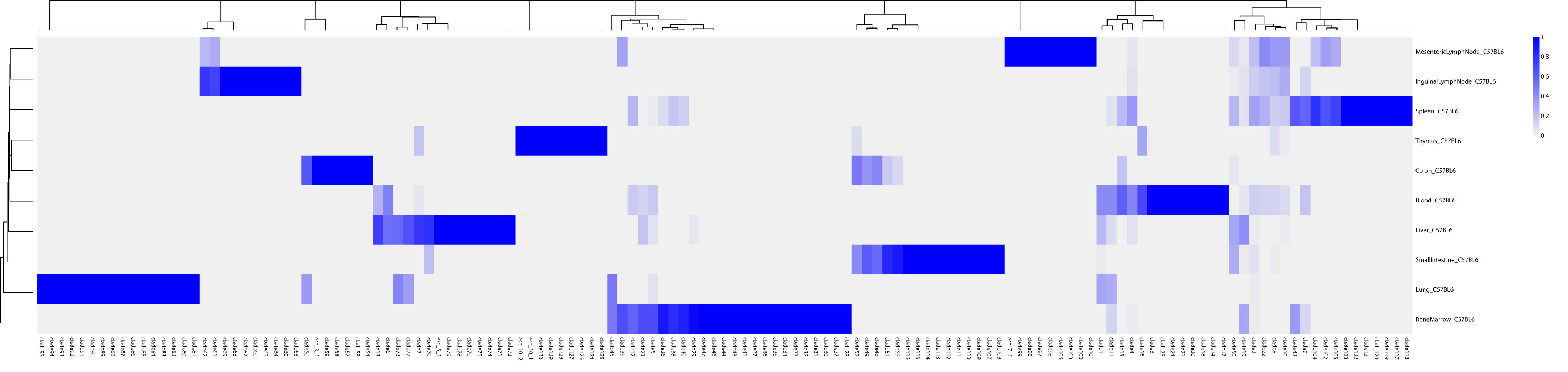

Supplement: S13 Fig — Higher resolution version of Fig 14 with clade labels. (TIF) [file pcbi.1005875.s013.tif]

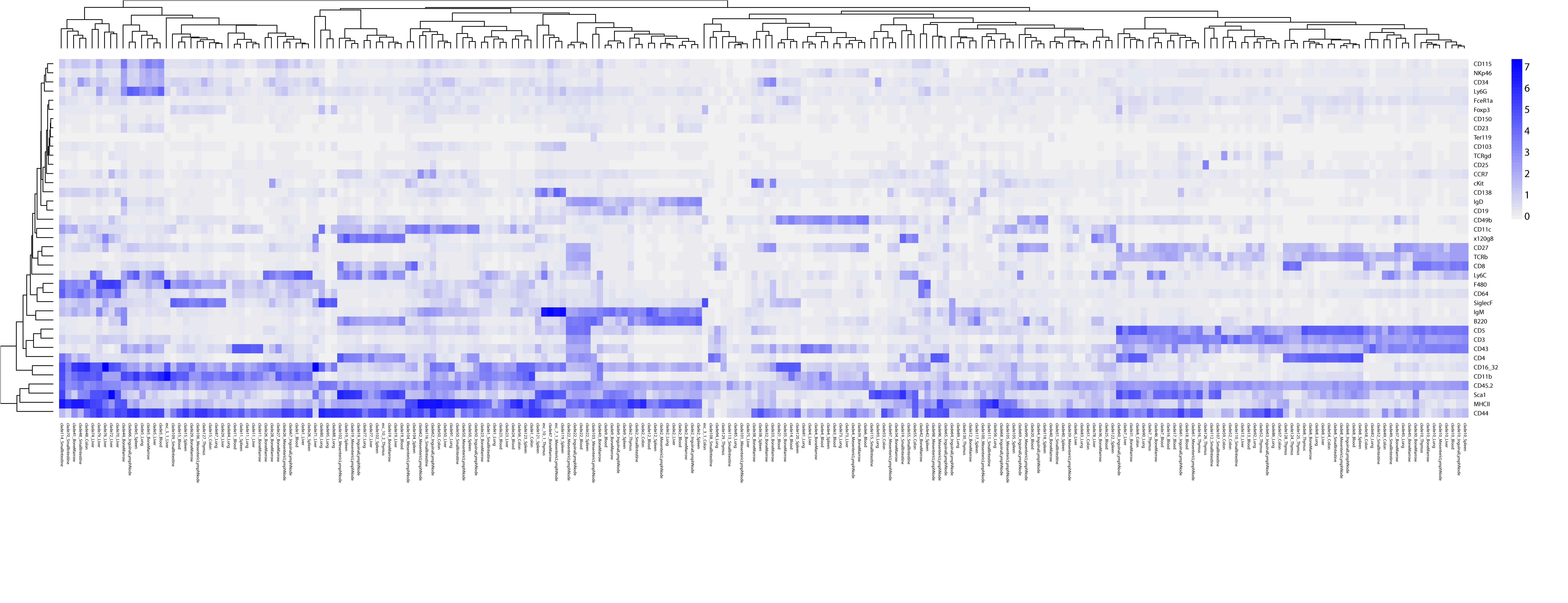

Supplement: S14 Fig — Higher resolution version of Fig 15 with clade labels. (TIF) [file pcbi.1005875.s014.tif]
